# The Effect of Probiotic Supplementation on Glucolipid Metabolism in Patients with Type 2 Diabetes: A Systematic Review and Meta-Analysis

**DOI:** 10.3390/nu15143240

**Published:** 2023-07-21

**Authors:** Rui Xiao, Linlin Wang, Peijun Tian, Xing Jin, Jianxin Zhao, Hao Zhang, Gang Wang, Minmin Zhu

**Affiliations:** 1 State Key Laboratory of Food Science and Resources, Jiangnan University, Wuxi 214122, China; 6210113228@stu.jiangnan.edu.cn (R.X.); wanglinlin@jiangnan.edu.cn (L.W.); pjtian@jiangnan.edu.cn (P.T.); 8202008362@jiangnan.edu.cn (X.J.); zhaojianxin@jiangnan.edu.cn (J.Z.); zhanghao61@jiangnan.edu.cn (H.Z.); 2 School of Food Science and Technology, Jiangnan University, Wuxi 214122, China; 3 National Engineering Research Center for Functional Food, Jiangnan University, Wuxi 214122, China; 4 (Yangzhou) Institute of Food Biotechnology, Jiangnan University, Yangzhou 225004, China; 5 Department of Anesthesiology and Pain Medicine, Jiangnan University Medical Center, Wuxi 214002, China

**Keywords:** type 2 diabetes mellitus, probiotics, glucolipid metabolism, randomised controlled trial

## Abstract

Purpose: Type 2 diabetes mellitus (T2DM) is a persistent metabolic condition with an unknown pathophysiology. Moreover, T2DM remains a serious health risk despite advances in medication and preventive care. Randomised controlled trials (RCTs) have provided evidence that probiotics may have positive effects on glucolipid metabolism. Therefore, we performed a meta-analysis of RCTs to measure the effect of probiotic therapy on glucolipid metabolism in patients with T2DM. Methods: With no constraints on the language used in the literature, Excerpta Medica Database, PubMed, the Cochrane Library, and the Web of Science were searched for pertinent RCTs published between the date of creation and 18 August 2022. Stringent inclusion and exclusion criteria were applied by two reviewers to independently examine the literature. The risk of bias associated with the inclusion of the original studies was assessed using the Cochrane risk-of-bias tool, and Stata 15.0 was used to perform the meta-analysis. Results: Thirty-seven publications containing a total of 2502 research participants were included in the meta-analysis. The results showed that after a probiotic intervention, the experimental group showed a significant decrease in body mass index (standardised mean difference (SMD) = −0.42, 95% confidence interval (CI) [−0.76, −0.08]), fasting glucose concentration (SMD = −0.73, 95% CI [−0.97, −0.48]), fasting insulin concentration (SMD = −0.67, 95% CI [−0.99, −0.36]), glycated haemoglobin concentration (SMD = −0.55, 95% CI [−0.75, −0.35]), Homeostatic Model Assessment for Insulin Resistance score (SMD = −0.88, 95% CI [−1.17, −0.59]), triglyceride concentration (SMD = −0.30, 95% CI [−0.43, −0.17]), total cholesterol concentration (SMD = −0.27, 95% CI [−0.43, −0.11]), and low-density lipoprotein concentration (SMD = −0.20, 95% CI [−0.37, −0.04]), and an increase in high-density lipoprotein concentration (SMD = 0.31, 95% CI [0.08, 0.54]). Moreover, subgroup analyses showed that patients with a longer intervention time, or those who were treated with multiple strains of probiotics, may benefit more than those with a shorter intervention time or those who were treated with a single probiotic strain, respectively. Conclusion: Probiotic supplementation improves glucolipid metabolism in patients with T2DM, offering an alternative approach for the treatment of these patients.

## 1. Introduction

It is estimated that more than 700 million people aged from 20 to 79 will have diabetes by 2045, with 90% of cases being type 2 diabetes mellitus (T2DM) [1]. Low-grade inflammation, B-cell depletion, and insulin resistance are the hallmarks of T2DM, which is a serious metabolic condition [2]. The main drugs used in the treatment of T2D at this stage are traditional insulin sensitizers such as biguanides and glitazones, traditional insulinotropic agents such as sulfonylureas and non-sulfonylureas, and newer insulinotropic agents such as glucagon-like peptide-1 receptor agonists and dipeptidyl peptidase-4 (DPP-4) inhibitors [3]. However, these medications have adverse effects. For instance, metformin may result in nausea, bloating, abdominal pain, vomiting, a deficiency of vitamin B12, and an unsettled stomach. Similar side effects, including indigestion, constipation, bloating, nausea, and vomiting, have been reported for liraglutide. These adverse effects are more likely to be caused by combination therapies than monotherapies [4]. Therefore, a safe monotherapy with few side effects is urgently needed to treat patients with T2DM.

An increased understanding of how gut microbiota influences metabolism has led to growing interest in the role that gut microbiota plays in the aetiology of T2DM [5,6]. Changes in the gut microbiota frequently accompany the onset and development of T2DM. For instance, compared with healthy individuals, patients with T2DM often have lower alpha diversity in their gut microbiota [7,8], in addition to a greater relative abundance of Gram-positive bacteria and actinomycetes, and a lower relative abundance of Gram-negative bacteria [9]. Given the strong relationship between host metabolism and gut bacteria, the composition of the gut microbiota offers an alternative target to guide the development of new anti-diabetic drugs. Probiotic supplementation has been shown to have beneficial effects regarding T2DM in numerous animal studies and randomised controlled trials (RCTs). For example, Ke et al. demonstrated that synbiotic interventions significantly decreased bodyweight and concentrations of fasting glucose, lipids, and other markers in mice with diet-induced obesity [10]. An RCT conducted by Sabico et al. showed that supplementation with a multi-strain probiotic decreased the insulin resistance index and concentrations of glycated haemoglobin and fasting glucose in individuals with T2DM [11]. Additionally, Kassaian et al. demonstrated that probiotics or synbiotics dramatically decreased concentrations of glycated haemoglobin in prediabetic patients [12].

Although several meta-analyses have found that probiotics alleviate the symptoms of T2DM, their clinical efficacy remains unclear because of the reviewed studies’ small sample sizes, high heterogeneity, high risk of bias, and suboptimal analytical methods [13,14,15,16].

Therefore, we followed the guidelines outlined in the Cochrane Handbook for Systematic Reviews of Interventions to perform a careful meta-analysis of how probiotics can be used to treat T2DM, with a focus on RCTs that were carried out in adults. Our aim was to identify the characteristics of potentially practical interventions—such as the number of strains used, the route of administration, the length of a course of therapy, and the probiotics’ ability to adapt to different physiological states of a host—and to assess the general clinical efficacy of probiotic supplementation for the treatment of T2DM.

## 2. Materials and Methods

The current inquiry was conducted in accordance with the Cochrane Handbook for the Systematic Review of Interventions and the Preferred Reporting Items for Systematic Reviews and Meta-Analyses. The PROSPERO database has this study protocol listed as ID CRD42023415503.

### 2.1. Inclusion Criteria

#### 2.1.1. Research Type

Randomised controlled trials.

#### 2.1.2. Research Subjects

(1) Aged 18 or older; (2) diagnosed with type 2 diabetes; (3) no allergy to probiotic components.

#### 2.1.3. Intervention Measures

Interventions: the test group received probiotics and the control group received a placebo.

#### 2.1.4. Outcome Indicators

The outcome indicators are HbA1c, fasting insulin, fasting glucose, HOMA-insulin resistance, TG, TC, LDL, and HDL.

### 2.2. Exclusion Criteria

(1) Non-related diseases; (2) study data could not be obtained; (3) non-randomized controlled trials, reviews, case reports, animal experiments, dissertations, or conference abstracts.

### 2.3. Retrieval Strategies

We incorporated mesh phrases and free keywords into our search strategy, in accordance with PICOS philosophy.

(1) Population (P): patients with T2DM.
(2) Intervention (I): probiotic supplementation.
(3) Comparison (C): placebo.
(4) Outcome (O): BMI, fasting glucose, fasting insulin, HbA1c, HOMA-insulin resistance, TG, TC, HDL, LDL.
(5) Study design (S): randomized clinical trials (RCTs).

For RCTs, regarding the impact of probiotics on T2DM, a thorough search of Web of Science, PubMed, Cochrane Library, and Embase was conducted. The data were retrieved, and they were published between the databases’ creation and 18 August 2022. The search strategy used a combination of subject terms and free words, with search terms including (“probiotic” OR “probiotics” OR “lactobacillus “ OR “ bifidobacterial “) AND (“diabetes” OR “glycemia” OR “glucose” OR “Fasting blood sugar” OR “FBS” OR “ Glycosylated haemoglobin A1c” OR “HbA1c” OR “insulin” OR insulin resistance “ OR “HOMA-IR” OR “cholesterol” OR “lipids” OR “Total cholesterol” OR “TC” OR “Triglyceride” OR “TG “ OR “High density lipoprotein cholesterol” OR “HDL-C” OR “Low density lipoprotein cholesterol” OR “LDL-C”, etc. There were no restrictions on language or region. The search strategy is detailed in Supplementary S1.

### 2.4. Literature Screening and Data Extraction

We used Endnote software, X9 edition, to manage all the documentation. We imported the literature into Endnote X9, and we filtered the original research so that it complied with the aims of this systematic evaluation. Then, we extracted information from the literature and double-checked it using unified quantity units. The subjects, first authors, publication year, nation, study type, sample size, and patient gender, in addition to the intervention method, intervention duration, and end indicators, comprise the bulk of the data gathered.

### 2.5. Risk of Bias Assessment

The included studies were assessed using the bias assessment tools suggested in the Cochrance Manual, including random sequence generation (selective bias), hidden bias distribution (selected biases), blinding of researchers and subjects (implemented bias), blind assessment of study results (measured biases), integrity of end data (follow-up bias), outcomes of selective reporting (report bias), and other sources (other bias).

### 2.6. Statistical Methods

Stata 15.0 software was used to statistically evaluate the included literature, and it also performed sensitivity analysis, publication bias analysis, and heterogeneity testing. Since the included data are continuous variables, and different qualitative pairings were used to measure the data in each study, the 95% confidence interval (CI) and standard mean difference (SMD) are combined. If *p* > 0.1 and *I*^2^ ≤ 50%, inter-study heterogeneity is accepted, and a fixed-effect model for meta-analysis is practicable. If *p* ≤ 0.1 or *I*^2^ > 50%, there is greater heterogenicity, and a random effect model for meta-analysis is practicable. All of the results are statistically significant, with a difference of *p* < 0.05 when the “metabias” command was used to publish the bias of the inclusion study.

## 3. Results

### 3.1. Literature Search Results

**Figure 1:**
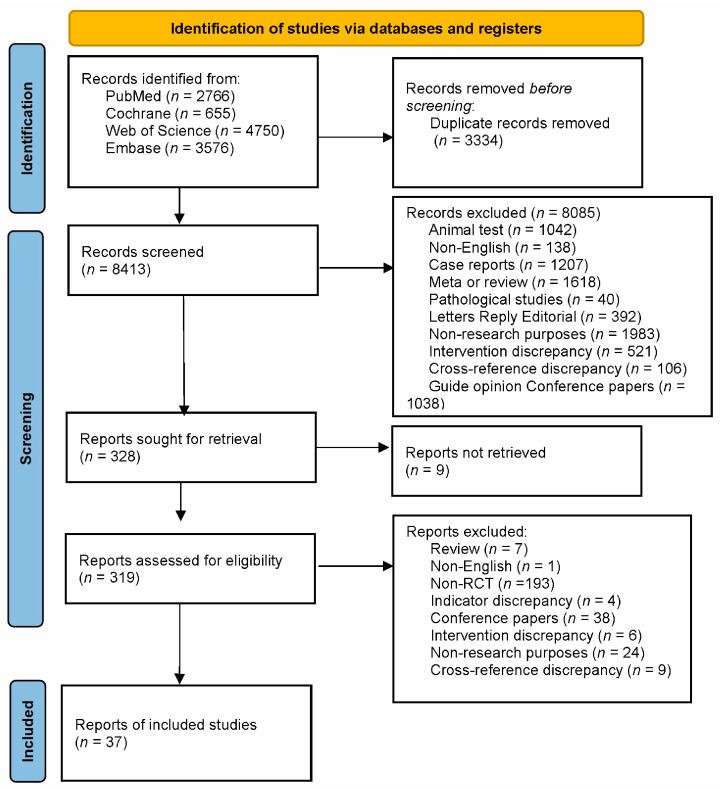
Flowchart of Study Selection.

The flowchart in Figure 1 shows the outcomes of the study selection procedure. In the original and additional searches, the aforementioned databases yielded 11,747 studies. The retrieved literature was imported into EndNote X9 for management, after which 3334 duplicate publications were eliminated, 8085 irrelevant publications were eliminated after reading the titles and abstracts, and 291 publications that did not meet the inclusion criteria were eliminated after reading the full text. This process yielded 37 studies for inclusion in this meta-analysis.

### 3.2. Basic Characteristics of the Included Studies

The 37 studies had English as their primary language and included 2502 patients (1262 in experimental groups and 1240 in control groups). Table 1 displays the key characteristics of these studies.

### 3.3. Quality Assessment of the Included Studies

**Figure 2:**
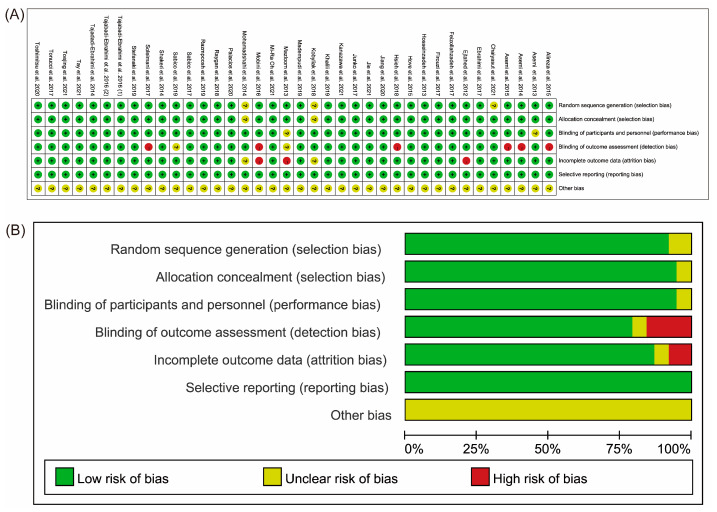
Bias risk evaluation for the studies that were included. The assessments concerning each risk of bias item for each included study are summarized in (**A**) of the risk of bias section. Low, high, and uncertain risk of bias are represented by the colours green, red, and yellow, respectively. (**B**) The risk of bias graph shows the percentages assigned to each risk of bias item across all included studies. Each point is assigned one of three bias assessment criteria, denoted by the colours green, red, or yellow, respectively: “Low”, “High”, and “Unclear” [11,17,18,19,20,21,22,23,24,26,27,28,29,30,31,32,33,34,35,36,37,38,39,40,41,42,43,44,45,46,47,48,49,50,51,52].

The included studies were all randomised, double-blind studies. Three of the included studies [34,35,40] did not mention whether random numbers were used for group allocation; two [34,35] did not mention whether allocation concealment was used; two [20,22] did not clarify whether blinding was used; eight [11,20,23,24,28,29,49,51] did not clarify whether there was measurement bias; and two [11,20] did not provide trial pre-registration or publication, therefore, it was not clear which outcome indicators should be reported. Moreover, five [20,26,29,34,35] studies had missing visit bias, and therefore, high reporting bias, but none of the other studies had reporting bias or any other biases. The results of the evaluation concerning the risk of bias in the included studies are shown in Figure 2.

### 3.4. Meta-Analysis Results

#### 3.4.1. Effects of Probiotic Therapy on Body Mass Index

**Figure 3:**
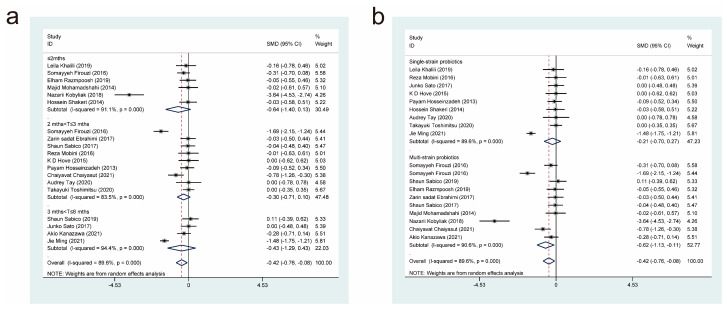
The impact of probiotic supplementation on BMI is shown using forest plots in a pooled study comparing against controls. The point estimate of the intervention effect is shown using solid black diamonds for each research item. The lower and upper bounds of the 95% CI for this effect are connected with the horizontal line. The subgroup and overall SMD, as determined using a random-effects model, are represented by the open diamonds. The red dotted line indicates pooled effect values. (**a**) shows a subgroup analysis of the duration of probiotic interventions, (**b**) shows a subgroup analysis of single or multiple probiotic strains [11,17,18,19,21,27,29,31,32,34,35,37,38,40,42,45,47,52].

Nineteen studies [11,17,18,19,21,27,29,31,32,34,35,37,38,40,42,45,47,52], with a total of 813 cases in the experimental group and 691 in the control group, examined the effects of probiotic therapy on body mass index (BMI) in patients with T2DM. Effect sizes were combined using a random effects model (*I*^2^ = 89.6%, *p* = 0.000), and the analysis revealed that the experimental group had a lower BMI than the control group (standardised mean difference (SMD) = −0.42, 95% confidence interval (CI) [−0.76, −0.08]; Figure 3).

We also conducted subgroup analyses on two intervention parameters, the duration of probiotic treatment (Figure 3a) and the bacterial strains used (Figure 3b), to further investigate whether probiotics have an effect on BMI in patients with T2DM. The subgroups had periods of probiotic treatment of equal to, or less than, 2 months (SMD = −0.64, 95% CI [−1.40, 0.13]), 2 months to 3 months (SMD = −0.30, 95% CI [−0.71, 0.10]), and 3 months to 6 months (SMD = −0.43, 95% CI [−0.43, 0.10]). It was found that none of the three periods of probiotic treatment were significantly different in terms of their effects on BMI (SMD = −0.43, 95% CI [−1.29, 0.43]). In the subgroup analysis based on different strains, the experimental interventions were more effective than the control interventions in reducing BMI when the former comprised multi-strain probiotics (SMD = −0.62, 95% CI (−1.13, −0.11]), but not when they comprised single-strain probiotics (SMD = −0.21, 95% CI [−0.70, 0.27]). Therefore, it can be inferred that treatment with multi-strain probiotics is superior to treatment with single-strain probiotics in terms of improving BMI in patients with T2DM.

#### 3.4.2. Effects of Probiotic Therapy on Fasting Blood Glucose Concentration

**Figure 4:**
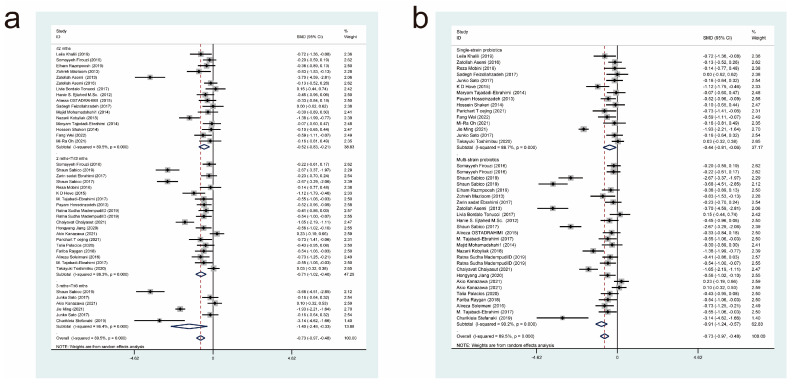
Impact of probiotic supplementation on fasting blood glucose levels is shown using forest plots in a pooled study comparing against controls. The point estimate of the intervention effect is shown using solid black diamonds for each research item. The lower and upper bounds of the 95% CI for this effect are connected with the horizontal line. The subgroup and overall SMD, as determined using a random-effects model, are represented by the open diamonds. The red dotted line indicates pooled effect values. (**a**) shows a subgroup analysis of the duration of probiotic interventions, (**b**) shows a subgroup analysis of single or multiple probiotic strains [11,17,18,19,20,21,22,23,24,26,27,28,29,30,31,32,33,34,35,36,37,38,39,40,41,42,43,44,45,46,47,48,49,50,51,52].

Thirty-seven studies [11,17,18,19,20,21,22,23,24,26,27,28,29,30,31,32,33,34,35,36,37,38,39,40,41,42,43,44,45,46,47,48,49,50,51,52] reported the effect of a probiotic intervention on fasting glucose concentration in patients with T2DM, and they were included in this analysis. After summarization, 1262 cases were included in the experimental group and 1240 cases were included in the control group. After combining effect sizes with a random-effects model (*I*^2^ = 89.5%, *p* = 0.000), it was revealed that compared with the control interventions, the experimental interventions significantly lowered fasting blood glucose concentrations (SMD = −0.73, 95% CI [−0.97, −0.48]; Figure 4).

Next, we performed subgroup analyses based on the duration of probiotic treatment (Figure 4a) and the strains used (Figure 4b). The subgroup analysis, based on the duration of probiotic treatment, showed that there were significant differences between probiotic treatment durations of less than or equal to 2 months (SMD = −0.52, 95% CI [−0.83, −0.21]), 2 to 3 months (SMD = −0.71, 95% CI [−1.02, −0.40]), and 3 to 6 months (SMD = −1.40, 95% CI [−2.48, −0.33]). It was evident that the longer the duration of administration, the more significantly probiotics decreased fasting blood glucose concentrations. The subgroup analysis based on the strains used showed that both multi-strain and single-strain probiotic treatments resulted in a significant reduction in fasting blood glucose concentrations compared with the control group, but multi-strain probiotic treatments (SMD = −0.91, 95% CI [−1.24, −0.57]) were more effective than single-strain probiotic treatments (SMD = −0.44, 95% CI [−0.81, −0.06]).

#### 3.4.3. Effects of Probiotic Therapy on Fasting Insulin Concentration

**Figure 5:**
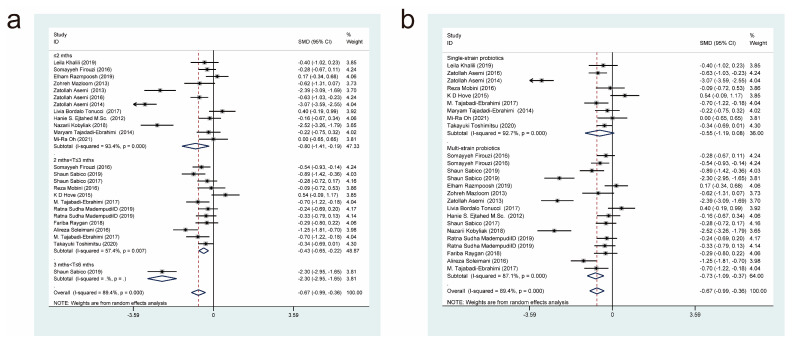
Impact of probiotic supplementation on fasting insulin levels is shown using forest plots in a pooled analysis comparing against controls. The point estimate of the intervention effect is shown using solid black diamonds for each research item. The lower and upper bounds of the 95% CI for this effect are connected with the horizontal line. The subgroup and overall SMD, as determined using a random-effects model, are represented by the open diamonds. The red dotted line indicates pooled effect values. (**a**) shows a subgroup analysis of the duration of probiotic interventions, (**b**) shows a subgroup analysis of single or multiple probiotic strains [11,17,18,19,20,22,23,24,25,26,27,29,30,32,33,35,36,39,44,45,47,49,50,51].

Twenty-five studies [11,17,18,19,20,22,23,24,25,26,27,29,30,32,33,35,36,39,44,45,47,49,50,51], with a total of 845 cases in the experimental group and 824 cases in the control group, reported the effect of probiotics on fasting insulin concentrations in patients with T2DM. A random-effects model (*I*^2^ = 89.4%, *p* = 0.000) was used to combine effect sizes, and the analysis showed that the experimental interventions had a more significant effect than the control interventions in terms of decreasing fasting insulin concentrations (SMD = −0.67, 95% CI [−0.99, −0.36]; Figure 5).

We also conducted subgroup analyses based on treatment duration (Figure 5a) and the probiotic strains used (Figure 5b). This revealed that intervention durations of less than or equal to 2 months (SMD = −0.80, 95% CI [−1.41, −0.19]), 2 to 3 months (SMD = −0.43, 95% CI [−0.65, −0.22]), and 3 to 6 months (SMD = −2.30, 95% CI [−0.99, −0.36]) resulted in significant reductions in fasting insulin concentrations. Multi-strain probiotics (SMD = −0.73, 95% CI [−1.09, −0.37]), but not single-strain probiotics (SMD = −0.55, 95% CI [−1.19, 0.08]), were found to be effective in reducing fasting insulin concentrations. This implies that multi-strain probiotics are superior to single-strain probiotics at lowering fasting insulin concentration in patients with T2DM.

#### 3.4.4. Effects of Probiotic Therapy on Glycated Haemoglobin Concentration

**Figure 6:**
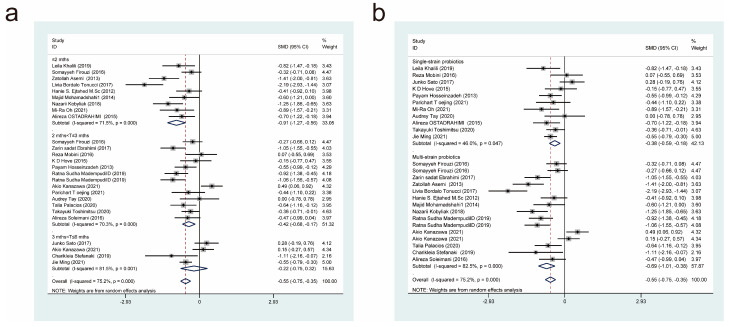
Impact of probiotic supplementation on glycosylated haemoglobin is shown using forest plots in a combined study comparing against controls. The point estimate of the intervention effect is shown using solid black diamonds for each research item. The lower and upper bounds of the 95% CI for this effect are connected with the horizontal line. The subgroup and overall SMD, as determined using a random-effects model, are represented by the open diamonds. The red dotted line indicates pooled effect values. (**a**) shows a subgroup analysis of the duration of probiotic interventions. (**b**) shows a subgroup analysis of single or multiple probiotic strains [17,18,21,22,25,26,29,31,32,33,34,35,37,39,42,43,44,45,46,47,48,49,51,52].

Twenty-six studies [17,18,21,22,25,26,29,31,32,33,34,35,37,39,42,43,44,45,46,47,48,49,51,52], with a total of 987 cases in the experimental group and 862 cases in the control group, reported the effect of a probiotic intervention on glycated haemoglobin concentration in patients with T2DM. The application of a random-effects model (*I*^2^ = 75.2%, *p* = 0.000) to combine effect sizes revealed that the experimental interventions were more efficacious than the control interventions in reducing glycated haemoglobin concentration (SMD = −0.55, 95% CI [−0.75, −0.35]; Figure 6).

We also conducted subgroup analyses based on the duration of probiotic treatment (Figure 6a) and the probiotic strains used (Figure 6b). Experimental intervention times of less than or equal to 2 months (SMD = −1.27, 95% CI [−1.27, 0.56]), 2 to 3 months (SMD = −0.42, 95% CI [−0.68, −0.17]), and 3 to 6 months (SMD = −0.22, 95% CI [−0.75, 0.32]) led to statistically significant differences in glycated haemoglobin concentration. Compared with the control interventions, both multi-strain and single-strain probiotic treatments resulted in significant reductions in glycated haemoglobin concentration, but multi-strain probiotic treatments (SMD = −0.69, 95% CI [−1.01, −0.38]) were more effective than single-strain probiotic treatments (SMD = −0.38, 95% CI [−0.59, −0.18]).

#### 3.4.5. Effects of Probiotic Therapy on Homeostatic Model Assessment for Insulin Resistance Score

**Figure 7:**
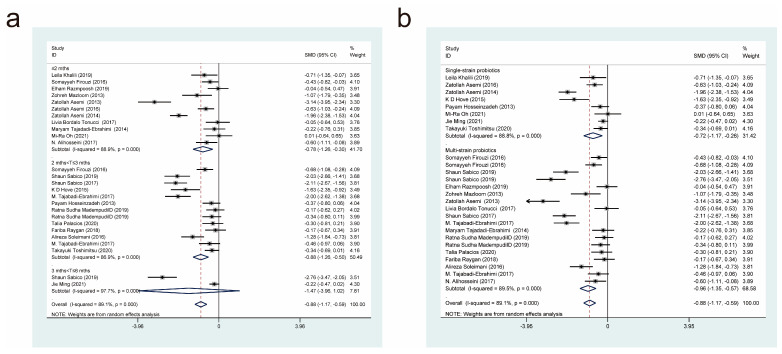
Impact of probiotic supplementation on HOMA-IR is shown using forest plots in a pooled study comparing against controls. The point estimate of the intervention effect is shown using solid black diamonds for each research item. The lower and upper bounds of the 95% CI for this effect are connected with the horizontal line. The subgroup and overall SMD, as determined using a random-effects model, are represented by the open diamonds. The red dotted line indicates pooled effect values. (**a**) shows a subgroup analysis of the duration of probiotic interventions, (**b**) shows a subgroup analysis of single or multiple probiotic strains [17,18,19,20,22,23,24,25,27,30,32,33,36,37,39,44,45,47,49,50,51,52].

Twenty-six studies [17,18,19,20,22,23,24,25,27,30,32,33,36,37,39,44,45,47,49,50,51,52], with a total of 1055 cases in the experimental group and 958 cases in the control group, examined the effect of probiotic interventions on the Homeostatic Model Assessment for Insulin Resistance (HOMA-IR) score in patients with T2DM. Combining effect sizes using a random-effects model (*I*^2^ = 89.1%, *p* = 0.000) revealed that the experimental interventions were more effective than the control interventions in lowering the HOMA-IR score (SMD = −0.88, 95% CI [−1.17, −0.59]; Figure 7).

We also conducted subgroup analyses based on the duration of probiotic treatment (Figure 7a) and the probiotic strains used (Figure 7b). Intervention durations of less than or equal to 2 months (SMD = −0.78, 95% CI [−1.26, 0.30]) and 2 to 3 months (SMD = −0.88, 95% CI [−1.26, −0.50]), but not 3 to 6 months (SMD = −1.47, 95% CI [−3.96, 1.02]), resulted in statistically significant effects on HOMA-IR scores. Compared with the control interventions, both multi-strain and single-strain probiotic treatments resulted in significant reductions in HOMA-IR scores, but multi-strain probiotic treatments (SMD = −0.96, 95% CI [−1.35, −0.57]) were superior to single-strain probiotic treatments (SMD = −0.72, 95% CI [−1.17, −0.26]).

#### 3.4.6. Effects of Probiotic Therapy on Triglyceride Concentration

**Figure 8:**
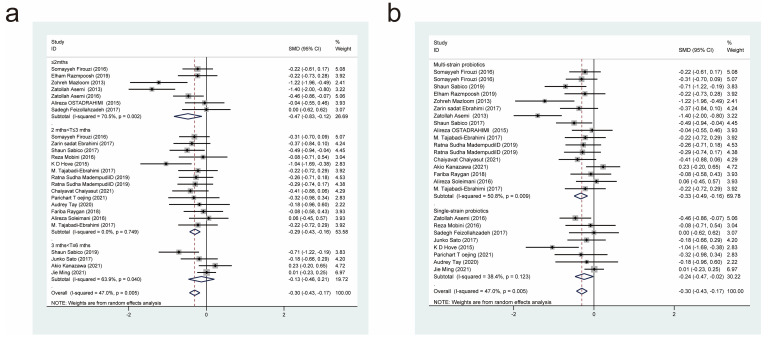
Impact of probiotic supplementation on TG is shown using forest plots in a pooled analysis comparing against controls. The point estimate of the intervention effect is shown using solid black diamonds for each research item. The lower and upper bounds of the 95% CI for this effect are connected with the horizontal line. The subgroup and overall SMD, as determined using a random-effects model, are represented by the open diamonds. The red dotted line indicates pooled effect values. (**a**) shows a subgroup analysis of the duration of probiotic interventions, (**b**) shows a subgroup analysis of single or multiple probiotic strains [11,18,19,20,21,22,23,24,27,28,29,30,31,32,33,39,40,42,43,45,49,50,51,52].

Twenty-five studies [11,18,19,20,21,22,23,24,27,28,29,30,31,32,33,39,40,42,43,45,49,50,51,52], with a total of 969 cases in the experimental group and 855 cases in the control group, examined the effect of probiotic interventions on triglyceride (TG) concentration in patients with T2DM. A random-effects model that combined effect sizes (*I*^2^ = 47%, *p* = 0.005) showed that the experimental interventions were more effective than the control interventions in decreasing TG concentration (SMD = −0.30, 95% CI [−0.43, −0.17]; Figure 8).

We also performed subgroup analyses based on the duration of probiotic treatment (Figure 8a) and the probiotic strains used (Figure 8b). Intervention durations of less than or equal to 2 months (SMD = −0.47, 95% CI [−0.83, 0.12]) and 2 to 3 months (SMD = −0.29, 95% CI [−0.43, −0.16]), but not 3 to 6 months (SMD = −0.13, 95% CI [−0.46, 0.21]), had statistically significant effects on TG concentrations. Compared with the control interventions, both multi-strain and single-strain probiotic treatments resulted in significant reductions in TG concentration, but multi-strain probiotic treatments (SMD = −0.33, 95% CI [−0.49, −0.16]) were more effective than single-strain probiotic treatments (SMD = −0.24, 95% CI [−0.47, −0.02]).

#### 3.4.7. Effects of Probiotic Therapy on Total Cholesterol Concentration

**Figure 9:**
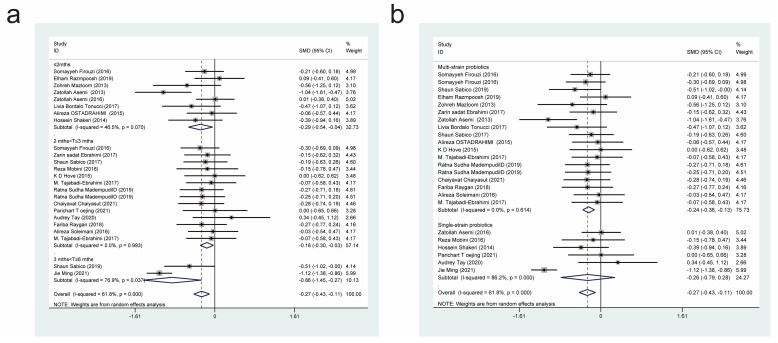
Impact of probiotic supplementation on TC is shown using forest plots in a pooled analysis comparing against controls. The point estimate of the intervention effect is shown using solid black diamonds for each research item. The lower and upper bounds of the 95% CI for this effect are connected with the horizontal line. The subgroup and overall SMD, as determined using a random-effects model, are represented by the open diamonds. The red dotted line indicates pooled effect values. (**a**) shows a subgroup analysis of the duration of probiotic interventions, (**b**) shows a subgroup analysis of single or multiple probiotic strains [11,18,19,20,21,22,23,24,25,27,28,29,30,32,33,38,39,40,43,45,49,50,51,52].

Twenty-four studies [11,18,19,20,21,22,23,24,25,27,28,29,30,32,33,38,39,40,43,45,49,50,51,52], with a total of 920 cases in the experimental group and 807 cases in the control group, examined the effect of probiotic interventions on total cholesterol (TC) concentration in patients with T2DM. A random-effects model that combined effect sizes (*I*^2^ = 61.8%, *p* = 0.000) showed that the experimental interventions were more effective than the control interventions in lowering TC concentration (SMD = −0.27, 95% CI [−0.43, −0.11]; Figure 9).

We also performed subgroup analyses based on the duration of probiotic treatment (Figure 9a) and the probiotic strains used (Figure 9b). Intervention durations of less than or equal to 2 months (SMD = −0.29, 95% CI [−0.54, −0.04]), 2 to 3 months (SMD = −0.16, 95% CI [−0.30, −0.03]), and 3 to 6 months (SMD = −0.86, 95% CI [−1.45, −0.27]) resulted in significant reductions in TC concentration. In the subgroup analysis based on different strains, the experimental interventions were more effective than the control interventions in reducing TC when the former comprised multi-strain probiotics (SMD = −0.24, 95% CI [−0.36, −0.13]), but not when they comprised single-strain probiotics (SMD = −0.26, 95% CI [−0.79, 0.28]).

#### 3.4.8. Effects of Probiotic Therapy on Low-Density Lipoprotein Concentration

**Figure 10:**
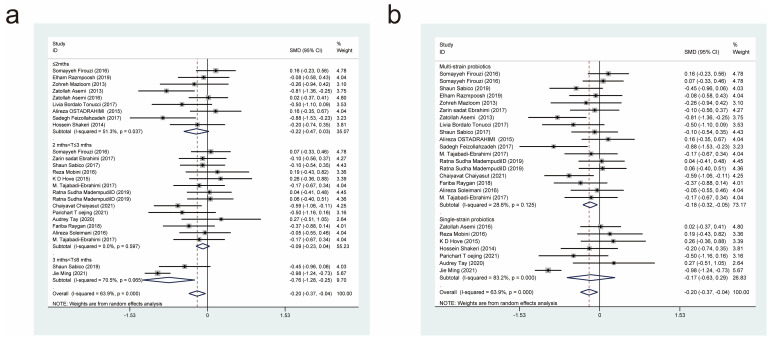
Impact of probiotic supplementation on LDL as measured by forest plots in a pooled analysis comparing against controls. The point estimate of the intervention effect is shown using solid black diamonds for each research item. The lower and upper bounds of the 95% CI for this effect are connected with the horizontal line. The subgroup and overall SMD, as determined using a random-effects model, are represented by the open diamonds. The red dotted line indicates pooled effect values. (**a**) shows a subgroup analysis of the duration of probiotic interventions, (**b**) shows a subgroup analysis of single or multiple probiotic strains [11,18,19,20,21,22,23,24,25,27,28,29,30,32,33,38,39,40,43,45,49,50,51,52].

Twenty-six studies [11,18,19,20,21,22,23,24,25,27,28,29,30,32,33,38,39,40,43,45,49,50,51,52], with a total of 1002 cases in the experimental group and 889 cases in the control group, examined the effect of probiotic interventions on low-density lipoprotein (LDL) concentration in patients with T2DM. Combining effect sizes using a random-effects model (*I*^2^ = 63.9%, *p* = 0.000) revealed that the experimental interventions were more effective than the control interventions in lowering LDL concentration (SMD = −0.20, 95% CI [−0.37, −0.04]; Figure 10).

We also performed subgroup analyses based on the duration of probiotic treatment (Figure 10a) and the probiotic strains used (Figure 10b). Intervention durations of less than or equal to 2 months (SMD = −0.22, 95% CI [−0.47, 0.03]) and 2 to 3 months (SMD = −0.09, 95% CI [−0.23, 0.04]) had no significant effect on LDL concentration. However, intervention durations of 3 to 6 months (SMD = −0.76, 95% CI [−1.28, −0.25]) had a significant effect on LDL concentration. In the subgroup analysis based on different strains, the experimental interventions were more effective than the control interventions in reducing LDL when the former comprised multi-strain probiotics (SMD = −0.18, 95% CI [−0.32, −0.05]), but not when they comprised single-strain probiotics (SMD = −0.17, 95% CI [−0.63, 0.29]).

#### 3.4.9. Effects of Probiotic Therapy on High-Density Lipoprotein Concentration

**Figure 11:**
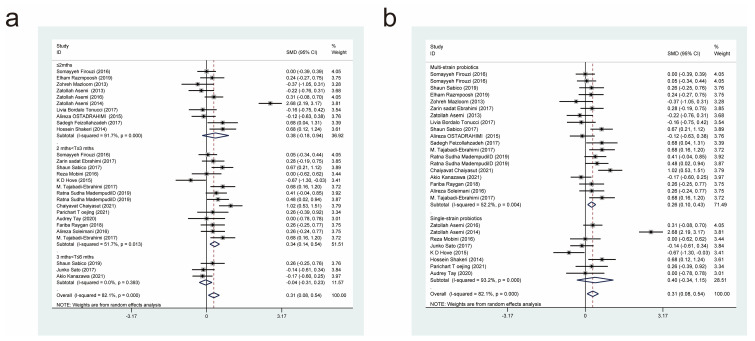
Impact of probiotic supplementation on HDL is shown using forest plots in a pooled study comparing against controls. The point estimate of the intervention effect is shown using solid black diamonds for each research item. The lower and upper bounds of the 95% CI for this effect are connected with the horizontal line. The subgroup and overall SMD, as determined using a random-effects model, are represented by the open diamonds. The red dotted line indicates pooled effect values. (**a**) shows a subgroup analysis of the duration of probiotic interventions, (**b**) shows a subgroup analysis of single or multiple probiotic strains [11,18,19,20,21,22,23,24,25,27,28,29,30,31,32,33,38,39,40,42,43,45,49,50,51,52].

Twenty-eight studies [11,18,19,20,21,22,23,24,25,27,28,29,30,31,32,33,38,39,40,42,43,45,49,50,51,52], with a total of 1080 cases in the experimental group and 965 cases in the control group, examined the effect of probiotic interventions on high-density lipoprotein (HDL) concentration in patients with T2DM. A random-effects model that combined effect sizes (*I*^2^ = 82.1%, *p* = 0.000) revealed that the experimental interventions were more effective than the control interventions in increasing HDL concentration (SMD = 0.31, 95% CI [0.08, 0.54]; Figure 11).

We also performed subgroup analyses based on the duration of probiotic treatment (Figure 11a) and the probiotic strains used (Figure 11b). An intervention duration of 2 to 3 months (SMD = 0.34, 95% CI [0.14, 0.54]) was effective in increasing HDL concentration, but those of less than or equal to 2 months (SMD = 0.38, 95% CI [−0.18, 0.94]), or 3 to 6 months (SMD = −0.04, 95% CI [−0.31, 0.23]), were not. In the subgroup analysis based on different strains, the experimental interventions were more effective than the control interventions in increasing HDL when the former comprised multi-strain probiotics (SMD = 0.26, 95% CI [0.10, 0.43]), but not when they comprised single-strain probiotics (SMD = 0.40, 95% CI [−0.34, 1.15]).

### 3.5. Sensitivity Analysis and Publication Bias Test

The included studies exhibited no sensitivity problems. However, Egger’s test was used to examine the funnel plots, and it demonstrated that there was publication bias for the HOMA-IR, TC, and TG data (*p* = 0.006, *p* = 0.003, and *p* = 0.02, respectively; Table 2). We employed funnel plots to depict publication bias, and we used the trim-and-fill method to evaluate these in light of the publication bias that existed in the included studies. Including eight studies in the TC model resulted in a symmetrical funnel plot, and the total effect size was −0.430 (−0.583, −0.277). No additional studies were required to make the funnel plots symmetrical for the IR and TG models, and the overall effect sizes were consistent (Table 3). Funnel plots and sensitivity analyses are provided in Supplementary S2.

## 4. Discussion

As the gut microbiota is crucial for preserving the body’s normal metabolism, it is now recognised as a significant ‘hidden’ organ in the human body [53]. The makeup of the gut microbiota varies significantly between individuals, and it is influenced by a several variables, including genetics, diet, lifestyle, and health status [54]. The composition of the gut microbiota has been linked to the occurrence and development of T2DM, and an imbalanced gut microbiota stimulates the body to produce cytokines that reduce insulin sensitivity and accelerate the onset of diabetes [55]. To control the balance of the gut microbiota and reduce insulin resistance in patients with T2DM, Zhai LX et al. have proposed probiotic administration [56]. Probiotics are microorganisms that change the gut microbiota of their host, and therefore, they have a variety of effects; for example, they can preserve the gut microbiota’s structural balance, increase the body’s antioxidant concentrations, and reduce intestinal inflammation [57]. Bianchi et al. found that probiotics can safely and efficiently change a patient’s gut microbiota, and they advised patients with metabolic illnesses to consume them frequently [58]. Lipopolysaccharides, which enter the blood to cause inflammation, disrupt intestinal integrity and influence the body’s glucose metabolism, primarily by increasing concentrations of glycated haemoglobin; they are much more prevalent in patients with T2DM than healthy individuals [59]. However, Kim YA et al. found that patients with T2DM who consume probiotics have significantly lower lipopolysaccharide concentrations, less endoplasmic reticulum stress, and improved insulin sensitivity [60].

In this meta-analysis, the effects of probiotics on glucolipid metabolism were assessed in 37 RCTs involving a total of 2502 participants with T2DM. Analysis of the combined data showed that after treatment, patients in the probiotic group had significantly lower fasting glucose and fasting insulin concentrations than those in the control group. This suggests that probiotics can assist patients with T2DM to control their blood sugar concentrations. Problems with gut microbiota cause patients with T2DM to have significantly lower glucagon-like peptide-1 concentrations than people without T2DM, which promotes gastric emptying in these patients and makes them feel hungry [61]. A hypoglycaemic effect can be produced via probiotic administration, as this promotes the production of glucagon-like peptide-1, thus inhibiting glucagon secretion and delaying gastric emptying [62]. This suggests that oral probiotics help to control glycated haemoglobin concentrations by promoting a glucagon-like peptide-1 release. More specifically, after treatment, glycated haemoglobin concentrations decreased to a significantly greater extent in the experimental group than in the control group. Additionally, the findings of this meta-analysis demonstrated that the change in HOMA-IR score after probiotic treatment was significantly greater in the experimental group than the control group. The HOMA-IR score reflects the equilibrium between hepatic glucose output and insulin secretion, allowing physicians to gauge the severity of a patient’s insulin resistance. The abovementioned finding implies that in patients with T2DM, oral probiotics may reduce insulin resistance and improve insulin sensitivity. However, publication bias was present in the meta-analysis of the HOMA-IR score (*p* = 0.006), which may be due to the calibre of the studies included.

Obesity is an independent risk factor for T2DM. Moreover, there are differences in the composition of the gut microbiota between obese and healthy patients, and disorders of the gut microbiota aggravate obesity [63,64]. Obese patients have elevated concentrations of cytokines, interleukins, the tumour necrosis factor, and lipopolysaccharides, resulting in a chronic inflammatory state, which leads to metabolic disorders and obesity-related diseases [65]. Oral probiotics can increase the abundance of certain intestinal microbiota to therefore alleviate metabolic syndrome problems and improve immune function in obese adults [66]. The results of this meta-analysis showed that compared with the control group, the experimental group exhibited significant improvements in BMI and in TG, TC, HDL, and LDL concentrations. However, there was publication bias in the meta-analysis of the TG and TC concentrations (*p* = 0.006) which may be related to the quality of the studies included. TG and HDL cholesterol concentrations can be controlled by altering the composition of the gut microbiota [67]. This could occur via several possible mechanisms, such as a reduction in the enterohepatic circulation of bile salts, the assimilation of cholesterol in the gastrointestinal tract, and the conversion of cholesterol to faecal sterols in the intestine [68]. In addition, the activation of farnesol receptors may also modulate TG concentrations [69], thereby improving lipid concentrations [70]. Mechanisms of cholesterol removal via lactic acid bacteria have been proposed in previous studies. For example, probiotics were found to purify bile salts by lowering cholesterol concentrations, due to the absorption of cholesterol by bacterial cell membranes via bile salt hydrolases [71]. Another study found that a probiotic mixture could modulate apolipoprotein synthesis via a mechanism that may be mediated by peroxisome proliferator-activated receptor/farnesoid X receptor upregulation during enterohepatic circulation [72]. Lactobacillus rhamnosus GG and the Bifidobacterium subfamily may partially prevent hepatic steatosis and damage by regulating the activation of hepatic adenylate-activated protein kinase [73,74]. Supplementation with Bifidobacterium shortum B-3 inhibits the accumulation of epithelial fat and upregulates the expression of genes related to lipid metabolism and insulin sensitivity [6]. The mechanisms of these effects may be diverse, and they need to be investigated further in vivo.

In accordance with our subgroup analysis, patients treated with multi-strain probiotics experienced greater reductions in fasting blood glucose concentrations than those treated with a single strain of probiotics. Chapman et al. observed that multi-strain probiotics combinations offer more health benefits than any constituent strain alone [75], which is consistent with our findings. Synergistic interactions between probiotic strains may account for this effect, and they may be the result of functional groups interacting with one another to enhance the host’s glycaemic parameters [6].

The following limitations apply to this meta-analysis. The probiotic doses varied significantly between the included studies, and some studies did not specify the doses used. Therefore, the effect of probiotic dose levels on glycaemic control in patients with T2DM requires confirmation in other studies. Guerrero-Bonmatty et al. showed that the combination of monacolin K (a statin) and L. plantarum strains was more effective in reducing LDL-C and TC levels in the treatment of hypercholesterolemia [76]. Therefore, in the future, some studies concerning probiotics in combination with statins, versus probiotics alone, could be considered. Moreover, general information about patients’ ages and BMIs varied between the included studies, which may have increased the clinical heterogeneity of the sample used for this meta-analysis.

In future research, there are many new probiotics, in addition to the traditional ones, worth considering for use at this stage. Since probiotics are excellent carriers or delivery devices, they can be recombined to orally deliver to antidiabetic targets [77]. Although probiotics are usually defined as living microorganisms, it might be worth considering whether dead strains are as potent, or even more potent, than live strains [78]. Although most of the probiotics that are widely used at this stage are lactic acid bacteria, there are also some potentially novel probiotics such as *Akkermansia muciniphila*, which also have antidiabetic properties [79]. In the future, further experiments and clinical trials will inevitably be needed to identify and compare the effects of different probiotic strains and dosages. Furthermore, the preparation technique and viability of probiotics, as well as the duration and regimen of good treatments, are also important factors that influence their antidiabetic activity.

## 5. Conclusions

In conclusion, this meta-analysis shows that oral probiotics aid in the regulation of glucolipid metabolism in patients with T2DM, primarily indicated by a marked decrease in glucose metabolism and lipid metabolism following treatment. These findings suggest that probiotic supplementation can be utilised as a complementary therapy to help prevent T2DM. To confirm the ability of probiotics to support glycaemic, lipid, and blood pressure regulation, additional clinical investigations with various patient profiles, probiotic dosages, and intervention durations are required.

## Figures and Tables

**Table 1:** Characteristics of the studies included in this meta-analysis.

Author/Year	Country	Intervention/Control (Size)	Age (Year)	Female/Male	Probiotic/Control	Dose (CFU/d)	Form	Duration	Measure Outcomes
Khalili et al., 2019 [17]	Iran	Probiotics (20)	43.95 ± 8.14	13/7	*Lacidophilus casei*	2 × 10^8^	Capsules	8 weeks	FBS/HbA1c/Insulin/HOMA-IR/ BMI
		Control (20)	45.00 ± 5.37	13/7	Maltodextrin				
Firouzi et al., 2017 [18]	Malaysia	Probiotics (68)	52.90 ± 9.20	NR	*L. acidophilus*, *L. casei*, *L. lactis*, Bifdobacterium, Actinobacteria, *B. bifdum*, *B.longum*, and *B. infantis*	6 × 10^10^	Powder	12 weeks	FBG/HbA1c/insulin/HOMA-IR/ TC/TG/HDL-C/LDL-C/BMI
		Control (68)	54.20 ± 8.30	NR	Placebo				
Sabico et al., 2019 [11]	Saudi Arabia	Probiotics (39)	48.00 ± 8.30	20/19	*B. bifdum* W23, *B. lactis* W52, *L. acidophilus* W37, *L. brevis* W63, *L. casei* W56, *L.salivarius* W24, *L. lactis* W19, and *L. lactis* W58	5 × 10^9^	Sachets	12 weeks	Glucose/Insulin/HOMA-IR/ TC/TG/HDL-C/LDL-C/BMI
		Control (39)	46.60 ± 5.90	18/21	Maize starch and maltodextrins				
Razmpoosh et al., 2019 [19]	Iran	Probiotic (30)	58.6 ± 6.5	13/17	*Lactobacillus acidophilus*, *Lactobacillus casei*, *Lactobacillus rhamnosus*, *Lactobacillus bulgaricus*, *Bifidobacterium breve*, *Bifidobacterium longum*, *Streptococcus thermophilus*	4.9 × 10^10^	Capsules	6 weeks	FBG/HbA1c/insulin/HOMA-IR/ TC/TG/BMI
		Control (30)	61.3 ± 5.2	14/16	Fructo-oligosaccharide, and Magnesium stearate				
Mazloom et al., 2013 [20]	Iran	Probiotic (16)	55.4	NR	*L. acidophilus*, *L. bulgaricus*, *L. bifidum*, and *L. casei*.	NR	Capsules	6 weeks	FBG/insulin/HOMA-IR/ TC/TG
		Control (18)	54.2	NR	Magnesium stearate				
Ebrahimi et al., 2017 [21]	Iran	Probiotic (35)	58.71 ± 8.20	12/23	*Lactobacillus* family, *Bifidobacterium* family, *Streptococus thermophilus*	NR	Capsules	9 weeks	FBG/TC/TG/HDL-C/LDL-C
		Control (35)	58.71 ± 8.20	16/19	Row starch				
Asemi et al., 2013 [22]	Iran	Probiotic (27)	50.51 ± 9.82	NR	*L. acidophilus*, *L. case*, *L. rhamnosus*, *L. bulgaricus*, *Bifidobacterium breve*, *B. longum*, *Streptococcus thermophilus*	3.92 × 10^10^	Capsules	8 weeks	FBG/HbA1c/insulin/HOMA-IR/ TC/TG/HDL-C/LDL-C/BMI
		Control (27)	52.59 ± 7.14	NR	Placebo				
Asemi et al., 2016 [23]	Iran	Probiotic (51)	NR	NR	*Lactobacillus sporogenes*	2.7 × 10^8^	Package	6 weeks	FBG/insulin/HOMA-IR/ TC/TG/HDL-C/LDL-C/BMI
		Control (51)	NR	NR	Isomalt, sorbitol and stevia				
Asemi et al., 2014 [24]	Iran	Probiotic (62)	NR	NR	*Lactobacillus sporogenes*	2.7 × 10^8^	Synbiotic food	6 weeks	FBG/insulin/HOMA-IR/ TC/TG/HDL-C/LDL-C/BMI
		Control (62)	NR	NR	Control food				
Tonucci et al., 2017 [25]	Brazil	Probiotic (23)	51.83 ± 6.64	11/12	*L. acidophilus* La-5 *B. lactis* BB-12	10^9^	Fermented goat milk	6 weeks	FBG/HbA1c/insulin/HOMA-IR/ TC/TG/HDL-C/LDL-C
		Control (22)	50.95 ± 7.20	8/14	Conventional fermented milk				
Ejtahed et al., 2012 [26]	Iran	Probiotic (30)	50.87 ± 7.68	19/11	*B. lactis* Bb12 and *L. acidophilus* La5	4 × 10^9^	Yogurt	6 weeks	FBG/HbA1c/insulin
		Control (30)	51.00 ±7.32	18/12	Conventional yogurts				
Sabico et al., 2017 [27]	Saudi Arabia	Probiotic (39)	48.0 ± 8.3	20/19	*B. bifdum* W23, *B. lactis* W52, *L. acidophilus* W37, *L. brevis* W63, *L. casei* W56, *L. salivarius* W24, *L. lactis* W19, and *L. lactis* W58	2 × 10^9^	Powder	12 weeks	FBG/insulin/HOMA-IR/ TC/TG/HDL-C/LDL-C
		Control (39)	46.6 ± 5.9	18/21	Maize starch and maltodextrins				
Alireza et al., 2015 [28]	Iran	Probiotic (30)	NR	12/18	Fermented milk (kefir) containing *L. casei*, *L. acidophilus*, and Bifidobacteria	4.6 × 10^10^	Fermented milk	8 weeks	FBG/HbA1c/TC/TG/HDL-C/ LDL-C
		Control (30)	NR	14/16	Fermented milk (dough)				
Mobini et al., 2016 [29]	Sweden	Probiotics (14)	64.00 ± 6.00	3/11	*L. reuteri* DSM 17938	10^8^ or 10^10^	Powder	12 weeks	FBG/HbA1c/insulin/TC/ TG/HDL-C/LDL-C
		Control (15)	65.00 ± 5.00	4/11	Placebo				
Feizollahzadeh et al., 2017 [30]	Iran	Probiotics (20)	56.9 ± 1.81	11/9	Soy milk containing *L. planetarum*	10^7^	Soy milk	8 weeks	FBS/TG/HDL-C/LDL-C
		Control (20)	53.60 ± 1.60	10/10	Conventional soy milk				
Junko et al., 2017 [31]	Japan	Probiotic (34)	64.0 ± 9.2	5/29	Lactobacillus casei strain Shirota-fermented milk	4 × 10^10^	Fermented milk	16 weeks	FBG/HbA1c/TC/TG/HDL-C/ BMI
		Control (34)	65.0 ± 8.3	14/20	Did not receive a probiotic				
Hove et al., 2015 [32]	Denmark	Probiotic (23)	58.5 ± 7.7	NR	L. helveticus Cardi04	NR	Yogurt	12 weeks	FBG/HbA1c/insulin/HOMA-IR/ TC/TG/HDL-C/LDL-C/BMI
		Control (18)	60.6 ± 5.2	NR	Artificially acidified milk				
Tajabadi-Ebrahimi et al., 2017 [33]	Iran	Probiotic (30)	64.20 ± 12.00	NR	*L. acidophilus*, *L. casei*, *L. lactis*, ifdobacterium, Actinobacteria, *B. bifdum*, *B.longum*, and *B. infantis*	2 × 10^9^	Capsule	12 weeks	FBG/HbA1c/insulin/HOMA-IR/ TC/TG/HDL-C/LDL-C
		Control (30)	64.00 ± 11.70	NR	Starch				
Mohamadshahi et al., 2014 [34]	Iran	Probiotic (22)	53.00 ±5.9	NR	*L. acidophilus* (La5) and *B. lactic* (Bb12)	1.1 × 10^9^	Yogurt	8 weeks	FBG/HbA1c
		Control (22)	49.00 ± 7.08	NR	Conventional yogurts				
Kobyliak et al., 2018 [35]	Ukraine	Probiotic (31)	52.23± 1.74	NR	LactobacillusþLactococcusþ Bifidobacteriumþ Propionibacteriumþ Acetobacter	10^12^	“Symbiter”	8 weeks	FBS/HbA1c/Insulin
		Control (22)	57.18± 2.06	NR	Placebos				
Tajadadi-Ebrahimi et al., 2014 [36]	Iran	Probiotic (27)	52.0 ± 7.2	NR	*L. sporogenes*	1 × 10^8^	Bread	8 weeks	FBG/insulin/HOMA-IR
		Control (27)	53.4 ± 7.5	NR	Control bread				
Hosseinzadeh et al., 2013 [37]	Iran	Probiotic (42)	46.8 ± 6.21	32/10	Brewer’s yeast	1.8 g	Tablets	12 weeks	FBS/HbA1c/HOMA-IR
		Control (42)	45.7 ± 6.11	31/11	Cellulose microcrystalline compounds, magnesium stearate, caramel, malt, and stearic acid.				
Shakeri et al., 2014 [38]	Iran	Probiotic (26)	52.3± 8.2	NR	*L. sporogenes*	1.2 × 10^10^	Breads	8 weeks	FBG/BMI/TC/TG/HDL-C/ LDL-C
		Control (26)	53.1± 7.5	NR	Control bread				
Madempudi et al., 2019 [39]	India	Probiotic (37)	53.60	7/30	*L. salivarius* UBLS22, *L. casei* UBLC42, *L. plantarum* UBLP40, *L. acidophilus* UBLA34, *B. breve* UBBr01, and *B. coagulans* Unique IS2	3 × 10^10^	Capsules	12 weeks	FBS/HbA1c/Insulin/HOMA-IR/TC/TG/HDL-C/ LDL-C
		Control (37)	50.50	9/28	Maltodextrin				
Chaiyasut et al., 2021 [40]	Thailand	Probiotic (36)	54.78 ± 1.92	NR	*Lactobacillus paracasei*, *Bifidobacterium longum*, Bifidobac-terium breve	10^11^	Sachet		FBG/BMI/TC/TG/HDL-C/ LDL-C
		Control (36)	58.94 ± 1.32	NR	Corn starch				
Jiang et al., 2020 [41]	China	Probiotic (42)	55.96 ± 8.45	27/15	*Bifidobacterium bifidum*, *Lactobacillus acidophilus*, *Streptococcus thermophilus*	9.7 × 10^9^	Sachet	12 weeks	FBS/HbA1c/Insulin/HOMA-IR
		Control (34)	56.12 ± 8.23	22/12	Starch				
Kanazawa et al., 2021 [42]	Japan	Probiotic (44)	61.1 ± 11.0	13/31	*Lacticaseibacillus paracasei* YIT 9029 (strain Shirota:LcS) organisms, *Bifidobacterium breve* YIT 12272	6 × 10^8^	Powder	24 weeks	FBS/HbA1c/TC/TG/HDL-C/ LDL-C
		Control (42)	55.9 ± 10.7	8/34	Placebo				
Toejing et al., 2021 [43]	Thailand	Probiotic (18)	63.50 ± 5.94	12/6	*L. paracasei*	5 × 10^10^	Powder	12 weeks	FBS/HbA1c/TC/TG/HDL-C/ LDL-C
		Control (18)	61.78 ± 7.73	16/2	Corn starch				
Mi-Ra Oh et al., 2021 [44]	Korea	Probiotic (20)	56.40 ± 11.57	14/6	*L. plantarum* HAC01.	4 × 10^9^	Capsules	8 weeks	FBS/HbA1c/Insulin/HOMA-IR
		Control (20)	53.55 ± 10.18	17/3	Microcrystalline cellulose				
Tay et al., 2021 [45]	New Zealand	Probiotic (15)	52.9 ± 8.7	9/6	*L. rhamnosus* HN001	6 × 10^9^	Capsules	12 weeks	FBS/HbA1c/TC/TG/HDL-C/ LDL-C
		Control (11)	54.1 ± 6.4	9/2	Microcrystalline cellulose and dextrose anhydrate				
Palacios et al., 2020 [46]	Australia	Probiotic (30)	61.4± 8.9	13/17	*Lactobacillus plantarum* Lp-115, *Lactobacillus bulgaricus* Lb-64, *Lactobacillus gasseri* Lg-36, *Bifidobacterium breve* Bb-03, *Bifidobacterium animalis* sbsp. lactis Bi-07, *Bifidobacterium bifidum* Bb-06, *Streptococcus thermophilus* St-21, *Saccharomyces boulardii* DBVPG 6763	5 × 10^10^	Capsules	12 weeks	FBS/HbA1c/Insulin/HOMA-IR
		Control (30)	56.1 ± 12.3	19/11	0 mg microcrystalline cellulose, 5 mg silica, and 10 mg magnesium stearate				
Toshimitsu et al., 2020 [47]	Japan	Probiotic (62)	50.6 ± 6.9	20/42	OLL2712	5 × 10^9^	Yogurt	12 weeks	FBS/HbA1c/Insulin/HOMA-IR
		Control (64)	51.2 ± 7.6	20/44	Placebo				
Stefanaki et al., 2019 [48]	Greece	Probiotic (7)	15	4/3	*Streptococcus thermophilus* (DSM24731), *Bifidobacteria breve* (DSM24732), *Bifidobacteria longum* (DSM2473), *Bifidobacteria infantis* (DSM24737), *Lactobacillus acidophilus* (DSM24735), *Lactobacillus plantarum* (DSM24730), *Lactobacillus paracasei* (DSM24733), *Lactobacillus delbreuckii* subspecies *bulgaricus* (DSM24734)	4.5 × 10^11^	Powder	24 weeks	FBS/TC/TG/HDL-C/LDL-C
		Control (10)	13.50	5/5	Placebo				
Hsieh et al., 2018 [49]	China Taiwan	Probiotic (22)	NR	NR	*L. reuteri*	4 × 10^9^	Capsules	24 weeks	FBS/HbA1c/Insulin/HOMA-IR/ TC/TG/HDL-C/LDL-C
		Control (22)	NR	NR	Placebo				
Raygan et al., 2018 [50]	Iran	Probiotic (30)	60.7 ± 9.4	NR	*Bifidobacterium bifidum*, *Lactobacillus casei*, *Lactobacillus acidophilus*	6 × 10^9^	Capsules	12 weeks	FBS/Insulin/HOMA-IR/ TC/TG/HDL-C/LDL-C/BMI
		Control (30)	61.8 ± 9.8	NR	Placebo				
Soleimani et al., 2017 [51]	Iran	Probiotic (30)	NR	10/20	*L. acidophilus*, *L. casei*, and *B. bifidum*	2 × 10^9^	Capsules	12 weeks	FBS/HbA1c/Insulin/HOMA-IR/ TC/TG/HDL-C/LDL-C/BMI
		Control (30)	NR	10/20	Placebo				
Jie et al., 2021 [52]	china	Probiotic (100)	54.16 ± 9.10	41/59	Bifidobacterium	2 × 10^8^	Capsules	16 weeks	FBS/HbA1c/TC/TG/HDL-C/ LDL-C/BMI
		Control (99)	52.73 ± 9.35	55/54	Lactose and magnesium stearate				

**Table 2:** Results of Egger’s test for publication bias.

Outcome Indicators	Items	Effect Size	Standard Error	95% CI	*t* Value	*p*
BMI	slope	−1.06756	0.6448588	(−2.37, 0.35)	−1.56	0.137
	bias	2.333851	2.712093	(−3.39, 8.06)	0.86	0.401
HDL	slope	0.6000644	0.7204764	(−0.88, 2.08)	0.83	0.413
	bias	−1.106745	2.812447	(−6.90, 4.69)	−0.39	0.697
HOMA-IR	slope	0.62113839	0.4490837	(−0.31, 1.55)	1.38	0.179
	bias	−0.58518	1.844773	(−9.39, −1.78)	−3.03	0.006
LDL	slope	−0.8541399	0.3113323	(−1.50, −0.21)	−2.74	0.012
	bias	2.536676	1.277253	(−0.11, 5.18)	1.99	0.059
TC	slope	−1.236445	0.2768925	(−1.81, −0.66)	−4.47	0.000
	bias	3.821232	1.143507	(1.45, 6.19)	3.34	0.003
TG	slope	0.2946404	0.2313827	(−0.18, 0.77)	1.27	0.216
	bias	−2.406254	0.9667479	(−4.41, −0.41)	−2.49	0.020
FBG	slope	0.3008114	0.4888035	(−0.69, 1.29)	0.62	0.542
	bias	−3.62217	1.900053	(−7.47, 0.22)	−1.91	0.064
Fasting insulin	slope	0. 5509178	0.7954488	(−1.09, 2.20)	0.69	0.496
	bias	−4.468728	3.06054	(−10.78, 1.86)	−1.46	0.158
glycosylated haemoglobin	slope	−0.0440752	0.3279199	(−0.72, 0.63)	−0.13	0.894
	bias	−1.885872	1.327238	(−4.63, 0.85)	−1.42	0.168

**Table 3:** Results of trim-and-fill method.

Outcome Indicators	Phase	Effect Size	95% CI	Z Value	*p*	No. of Studies
HOMA-IR	Before	−0.879	(−1.169, −0.589)	−5.944	0	26
	After	−0.879	(−1.169, −0.589)	−5.944	0	26
TC	Before	−0.267	(−0.429, −0.105)	−3.235	0.001	24
	After	−0.430	(−0.583, −0. 277)	−5.494	0.000	32
TG	Before	−0.301	(−0. 435, −0.167)	−4.413	0.000	25
	After	−0.301	(−0. 435, −0.167)	−4.413	0.000	25

## Data Availability

No new data were created or analyzed in this study. Data sharing is not applicable to this article.

## Supplementary Materials

The following supporting information can be downloaded at: https://www.mdpi.com/article/10.3390/nu15143240/s1, Supplementary S1: Details of searching strategy and screening process. Supplementary S2: funnel plots and sensitivity analysis.
