# Supplementary material for: The Effect of Probiotic Supplementation on Glucolipid Metabolism in Patients with Type 2 Diabetes: A Systematic Review and Meta-Analysis
Source: Nutrients. 2023 Jul 21;15(14):3240. doi: 10.3390/nu15143240 (PMC10383415; doi:10.3390/nu15143240)
Supplement: Supplementary file 1 [file nutrients-15-03240-s001.zip › Supplementary S1 Details of searching strategy and screening process.pdf]

Supplementary S1 Details of searching strategy and screening process

WOS

Search strategy: **((((((("Diabetes Mellitus"[Mesh]) OR (diabetes[Title/Abstract])) OR (Diabetes Mellitus[Title/Abstract])) OR (diabete[Title/Abstract])) OR (diabetic[Title/Abstract])) OR (hyperglycemia[Title/Abstract])) OR (Glucose Intolerance[Title/Abstract])) AND ((((("Probiotics"[Mesh]) OR (probiotics[Title/Abstract])) OR (probiotic[Title/Abstract])) OR (lactobacillus[Title/Abstract])) OR (bifidobacterial[Title/Abstract]))**

Search results:4750

PubMed

Search strategy: **((((((("Diabetes Mellitus"[Mesh]) OR (diabetes[Title/Abstract])) OR (Diabetes Mellitus[Title/Abstract])) OR (diabete[Title/Abstract])) OR (diabetic[Title/Abstract])) OR (hyperglycemia[Title/Abstract])) OR (Glucose Intolerance[Title/Abstract])) AND ((((("Probiotics"[Mesh]) OR (probiotics[Title/Abstract])) OR (probiotic[Title/Abstract])) OR (lactobacillus[Title/Abstract])) OR (bifidobacterial[Title/Abstract]))**

Search results:2766

Cochrane

#1 MeSH descriptor: [Diabetes Mellitus] explode all trees 35497

#2 (Glucose Intolerance):ti,ab,kw OR (diabetes):ti,ab,kw OR (diabete):ti,ab,kw OR (diabetic):ti,ab,kw OR (hyperglycemia):ti,ab,kw 109291

#3 #1 or #2 109492

#4 MeSH descriptor: [Probiotics] explode all trees 2563

#5 (probiotics):ti,ab,kw OR (probiotic):ti,ab,kw OR (lactobacillus):ti,ab,kw OR (bifidobacterial):ti,ab,kw 10187

#6 #4 or #5 10232

#7 #3 and #6 655（Trials 647）

Embase

Embase Session Results

No.	Query	Results
**#3**	**#1** AND **#2**	3,576
**#2**	**'probiotics'**/exp OR **probiotics** OR **probiotics**:ti,ab,kw OR **probiotic**:ti,ab,kw OR **lactobacillus**:ti,ab,kw OR **bifidobacterial**:ti,ab,kw	84,882
**#1**	**'diabetes mellitus'**/exp OR **'diabetes mellitus'** OR ((**'diabetes'**/exp OR **diabetes**) AND **mellitus**) OR **'diabetes mellitus'**:ti,ab,kw OR **diabetes**:ti,ab,kw OR **diabete**:ti,ab,kw OR **diabetic**:ti,ab,kw OR **hyperglycemia**:ti,ab,kw OR **'glucose intolerance'**:ti,ab,kw	1,390,312
