# Supplementary material for: The Effect of Probiotic Supplementation on Glucolipid Metabolism in Patients with Type 2 Diabetes: A Systematic Review and Meta-Analysis
Source: Nutrients. 2023 Jul 21;15(14):3240. doi: 10.3390/nu15143240 (PMC10383415; doi:10.3390/nu15143240)
Supplement: Supplementary file 1 [file nutrients-15-03240-s001.zip › Supplementary S2 funnel plots and sensitivity analysis/funnel plots.zip!/funnel plots/fasting blood glucose.pdf]

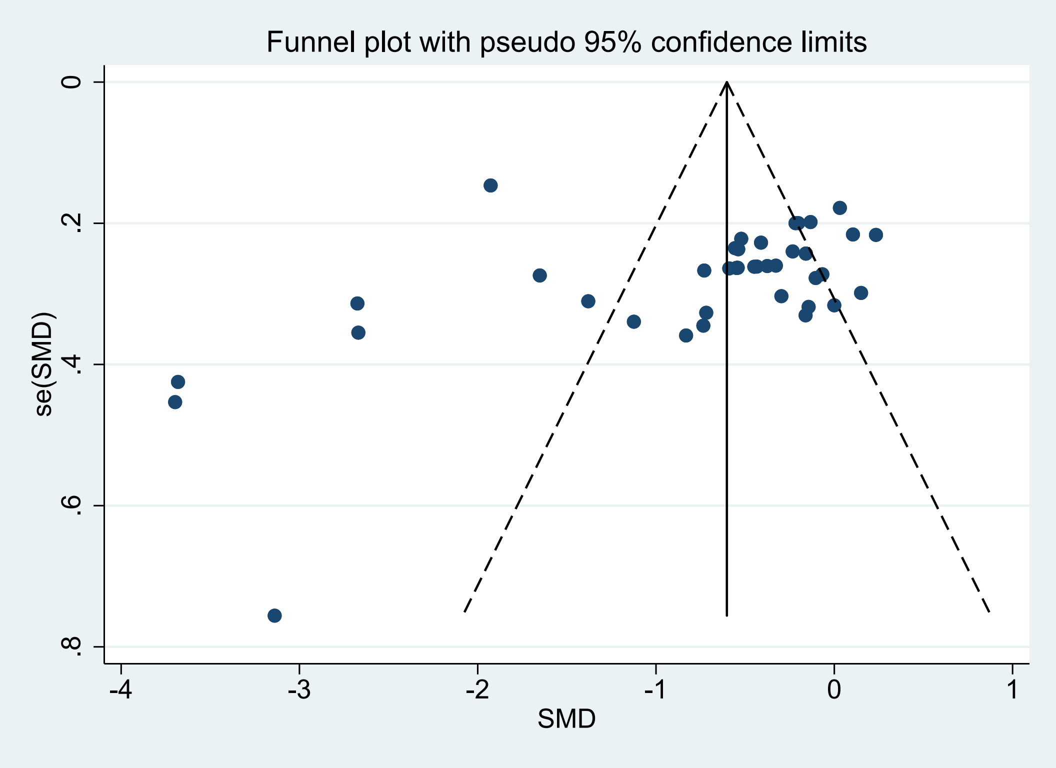
Funnel plot with pseudo 95% confidence limits
se(SMD)
0
.2
.4
.6
.8
-4
-3
-2
-1
0
1
SMD
